# Fluorescein and Indocyanine Green Angiography for Uveitis

**DOI:** 10.4103/0974-9233.58419

**Published:** 2009

**Authors:** Carl P Herbort

**Affiliations:** Department of Ophthalmology, Retinal and Inflammatory Eye Diseases, Centre for Ophthalmic Specialized Care and University of Lausanne, Switzerland

**Keywords:** Fluorescein, Indocyanine Green Angiography, Uveitis

## Abstract

In recent years enormous progress has been achieved in investigational procedures for uveitis. Imaging is one such example with the advent of new methods such as indocyanine green angiography, ultrasound biomicroscopy and optical coherence tomography to cite only the most important. This tremendous increase in precision and accuracy in the assessment of the level and degree of inflammation and its monitoring comes in parallel with the development of extremely potent and efficacious therapies. In view of these developments, our whole attitude in the appraisal and investigation of the uveitis patient has to be adapted and correctly reoriented integrating the recent developments and this is no different for ocular angiography.

## INTRODUCTION

**Figure 1:**
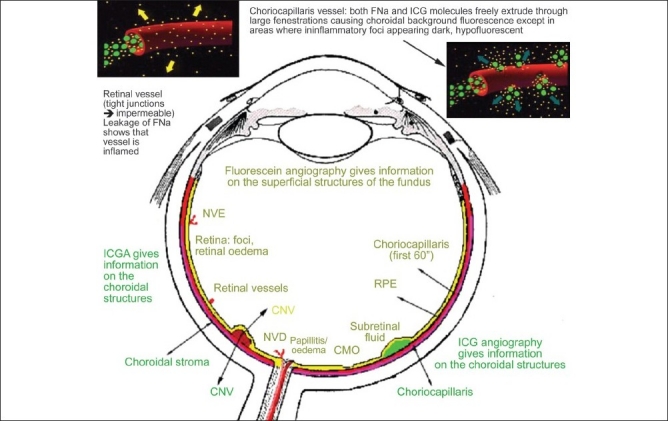
Complementary information from fluorescein fundus angiography and from indocyanine green angiography FFA draws its advantages from the small size of the fluorescein molecule (picture top left) and gives information on all superficial structures of the fundus including preretinal and intraretinal hemorrhages, optic disc, retinal vessels, neovessels, retina, macula, sub retinal space, RPE and chorioretinal atrophy, choriocapillaris and choroidal neovessels. ICGA draws its advantage from the infrared fluorescence of the ICG molecule and from its macromolecular behavior. ICG extrudes from the largely fenestrated choriocapillaris (picture top right) and impregnates and is stuck in the choroidal stroma, so low lighting inflammatory foci seen in dark because diffusion of ICG is impaired CMO - cystoid macular oedema; CNV - choroidal neovessels; NVD - neovessels disc; NVE - neovessels elsewhere; RPE - retinal pigment epithelium

One of the essential procedures performed in uveitis to complement clinical appraisal of the patient with intraocular inflammation is angiographic investigation of the posterior segment. Angiography may be performed to confirm elements already revealed by clinical examination or other investigational methods such optical coherence tomography (OCT). A second reason to perform an angiography is for better grading of the inflammation of the fundus. A third reason is to make a good baseline inventory of inflammatory involvement to subsequently use it for follow-up purposes. In follow-up situations, angiography is usually performed to monitor disease intensity and impact of therapy [Figure 1].

Fluorescein angiography (FFA) is being performed for over 40 years. As fluorescein sodium fluoresces in the wavelengths of the visible light, they mainly give information on the superficial structures of the fundus and therefore, mostly, only confirm signs already known to the clinician as in most instances OCT is already available when FFA is performed.

Since about 15 years, a second angiographic procedure is being used, using indocyanine green, a dye that fluoresces in the infrared wavelengths. It allows imaging of the poorly accessible choroid beforehand. This procedure, in contrast to FFA, often gives additional information undetected by clinical examination or FFA or OCT. Therefore, indocyanine green angiography (ICGA) is indispensable in the proper assessment of inflammatory involvement in uveitis as it gives information, which is otherwise lost. Very often, ICGA has a diagnostic value, rarely the case for FFA. For all these reasons, in most cases where angiographic work-up is required and choroidal involvement cannot be excluded, dual FFA and ICGA should be performed.^1^–^4^

## FUNDUS FLUORESCEIN ANGIOGRAPHY PRINCIPLES

### The molecule: Fluorescein sodium (FNa)

In fluorescein angiography, a natural dye, fluorescein sodium (FNa) is injected intravenously to analyze the blood circulation of the ocular fundus, mainly the retina. Fluorescein sodium is a small hydrosoluble molécule of 354 daltons of which 80% is bound to proteins and 20% is free, the latter free form being responsible for the emission of fluorescing light.

Two crucial principles characterize FNa. Firstly, fluorescein has a micromolecular behavior. This means that fluorescein easily gets out, at the slightest breakdown of the hemato-retinal barrier, from the usually impermeable retinal vessels. It diffuses easily into tissues but is also quickly washed out. The other characteristic is that fluorescein fluoresces at 520–530 nanometers within the wavelengths of visible light and is, therefore, blocked by the retinal pigment epithelium (RPE), giving no useful information on choroidal circulation and compartment except on the choriocapillaris during the first 40–60″ of angiography. These characteristics determine the use and limitations of fluorescein angiography.

### FFA signs

**Figure 2:**
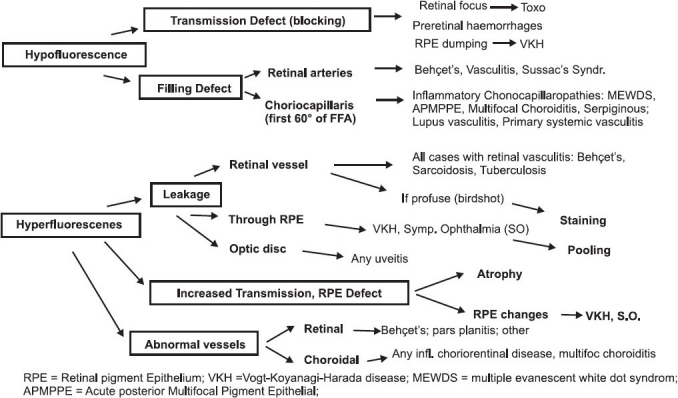
Schematic diagram of fluorescein angiographic signs in uveitis

Fluorescein angiographic concepts, classically described since more than 40 years, also apply to inflammatory diseases [Figure 2]. Increased fluorescence can be due to three main mechanisms: (1) leakage producing pooling (in a space) or staining (in tissues); (2) increased transmission of fluorescence due to fundus atrophy with removal of the RPE producing larger hyperfluorescent areas or due to smaller window-defects produced by areas of RPE defects; (3) presence of abnormal vessels (retinal vessels or choroidal neovascular membranes). Decreased fluorescence can be either due to transmission decrease (blockage) or filling defect (vascular delayed perfusion or non perfusion).

### Fluorescein angiography performed for uveitis: Methodological aspects

It is beyond the scope of this article to describe the equipment and instruments used. However, there are some adjustments of the angiographic timing and technique for uveitis to be observed. Early frames are important and should be taken up to two to three minutes from the posterior pole as information from the choriocapillaris is available only up to about 60 seconds and will indicate whether there is any choriocapillaris filling defect. Regarding the retinal circulation, early frames give us information on the arteriovenous circulation time, the presence of disc neovessels (NVD), and presence of diffuse capillary leakage. At five to seven minutes, full 360 degree panoramas are performed and thereafter (10–12 minutes), again, posterior pole frames are taken and frames of specific lesions identified during previous angiograsphic times.

## FFA SYMPTOMATOLOGY AND USE FOR UVEITIS

The clinician should know what information he can expect from FFA and be aware that superficial structures such as the optic nerve head, retinal vessels, retina and sub retinal space will be highlighted by FFA. In addition, FFA is an ideal tool to analyze the RPE and atrophic areas that can be seen, thanks to the underlying fluorescence seen through window defects of the RPE or due to complete absence of the RPE screen in chorioretinal atrophy. FFA is an inadequate method to analyze the choroid as the RPE is a screen for the choroidal fluorescence. However, during early FFA frames (up to 60″), because early choroidal fluorescence is so strong, information on the choriocapillaris is also obtained. Moreover, choroidal fluorescence is at the base of the two main FFA concepts of “window defect” when choroidal fluorescence is seen through areas of missing RPE and the concept of “masking (blocking) effect” in which choroidal fluorescein background fluorescence is attenuated or lost due to decreased transmission, which can be seen in individuals with African ancestry.

The FFA signs are not analyzed according to angiographic principles exposed in Figure 1, but according to the anatomical structure, going antero-posteriorly from optic disc to choriocapillaris.

Before analyzing the different structures, it should be specified that some posterior uveitis can cause retinal or preretinal hemorrhages producing hypofluorescence through a “blocking effect”.

### Optic disc

**Figure 3:**
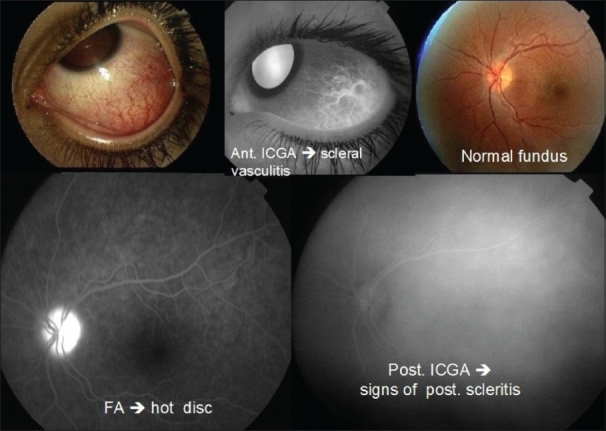
Case of anterior scleritis (top left) with vasculitis shown by anterior ICGA (top middle) with no apparent clinical posterior involvement seen on fundus examination (top right). FFA clearly shows hyperfluorescent hot disc indicating posterior involvement (bottom left) which was confirmed by ICGA showing diffuse choroidal hyperfluorescence confirming the presence of posterior scleritis (bottom right)

FFA is an adequate and sensitive method to detect inflammation of the optic discs which appear hyperfluorescent and, in addition, which leak in case of severe inflammation. FFA is especially helpful when clinical examination does not clearly reveal inflammation of the optic discs. Subclinical papillitis (hyperfluorescence) can be detected by FFA and this is useful in conditions known to be bilateral when the controlateral, apparently uninvolved eye, shows angiographic papillitis. FFA usually helps differentiate papillitis from papilloedma. In both conditions there are early dilated capillaries and late hyperfluorescence. The papilla is, however, more swollen in papilloedema than in papillitis. Papillitis is more prone to produce leakage [Figure 3].

### Retinal vessels

FFA furnishes three types of information on the retinal circulation which include: (1) imaging of inflammatory damage to vessel walls in retinal vasculitis, (2) display occlusive vasculopathy of retinal arteries or arterioles and (3) detection of retinal neovessels situated at the disc (NVD) or elsewhere in the retina (NVE).

#### Retinal vasculitis or inflammatory vasculopathy

**Figure 4:**
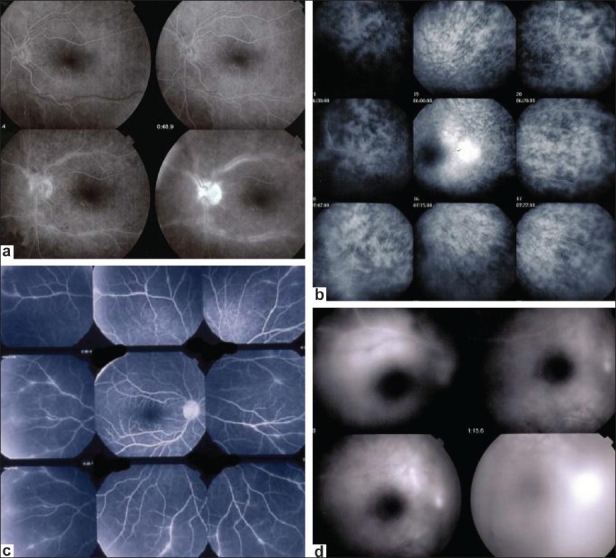
FFA signs: Retinal vasculitis; (a) Birdshot retinochoroidopathy; (b) Severe vasculitis in Behçet's uveitis; (c) Vasculitis in intermediate uveitis related to multiple sclerosis; (d) Disc neovessels and retinal neovessels in intermediate uveitis of the pars planitis type

Retinal vessels normally are highly impermeable due to the tight junctions between endothelial cells and they usually don't leak. However, the slightest inflammation of retinal vessel walls allows the small fluorescein molecule (359 daltons) to leak out of vessels and FFA is therefore a very sensitive to detect retinal vessel inflammation mostly veins [Figure 1]. Leakage is a characteristic of the disease for Behçet's uveitis, intermediate uveitis of the pars planitis type, intermediate uveitis related to multiple sclerosis, intermediate uveitis of unknown cause and birdshot chorioretinopahty [Figure 4]. However, every inflammatory focus of the fundus will have areas of leaking vessels in its vicinity. A special situation is represented by the particular vasculopathy of frosted branch angiitis with thick perivascular sheathing not only visible on FFA but also on fundus examination. Frosted branch angiitis can be associated with herpetic or cytomegalovirus, especially in the context of AIDS, as well as with toxoplamic retinitis, SLE, Crohn's disease and leukemia and lymphomas.^5^

#### Occlusive retinal vasculopathy

**Figure 5:**
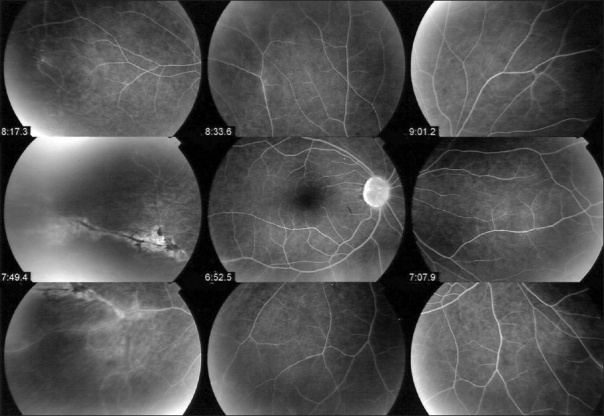
Retinal non perfusion in a case of Behçet's uveitis in temporal inferior area

Areas of non perfusion are best investigated with FFA that shows the extent of retina involved due to ischemia. Inflammatory conditions that often present retinal non perfusion include, without being exhaustive, Behçet's uveitis [Figure 5], sarcoidosis, systemic lupus erythematosus (SLE), Eales' disease or more appropriately called tuberculous hypersensitivity retinal vasculitis, IRVAN (Idiopathic retinal vasculitis, aneurisma and neuro-retinitis) and Susac's syndrome.

Retinal neovascularization is best demonstrated by fundus fluorescein angiography (FFA) and can complicate any of the conditions that produce retinal non perfusion^6^ [Figure 4d].

### Retina

#### Peripheral retina

**Figure 6:**
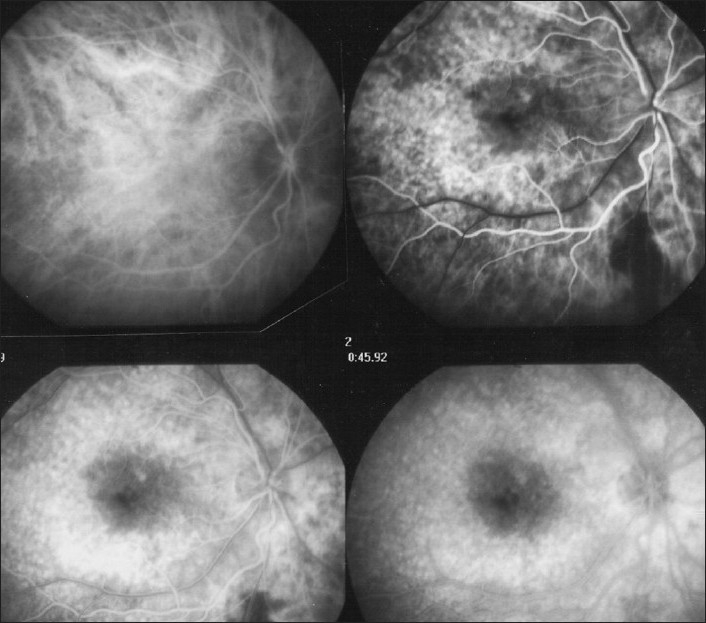
Birdshot chorioretinopathy. Birdshot is an inflammatory disease where the choroid and retina are primary independent targets of the inflammatory process. Retinal involvement can show profuse capillary leakage causing massive exudation into the retina at the origin of a pseudo-delay of arteriovenous circulation as large veins are still not opacified at 45 seconds (bottom right frame). The ICG frame (top left) shows that arteriovenous transit is already complete at 19 seconds

FFA is the investigation of choice of retinal foci that appear hypofluorescent in the early phases and progressively become hyperfluorescent. Some conditions such as birdshot chorioretinopathy produce profuse capillary leakage that produces bright and diffuse hyperfluorescence of the whole retina [Figure 4]. The capillary leakage is sometimes such that there is not enough dye to mark the large veins. Gass interpreted this angiographic finding as perfusion delay. Indocyanine green angiography has helped us understand that the arteriovenous perfusion time is normal^7^,^8^ [Figure 6].

#### Macula

**Figure 7:**
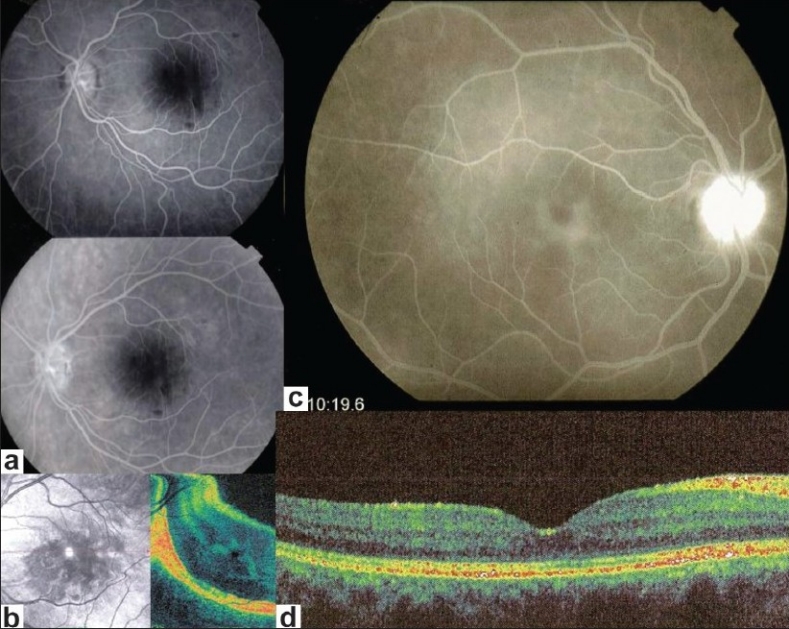
FFA signs: Macular ischemia and macular oedema; (a and b) FFA (7a) of macula showing central hypofluorescence (enlargement of avascular zone) indicating macular ischemia; change in tissue density well shown on a C-scan OCT (7c, right picture. Delineation of ischemia is clearly shown on SLO fundus picture (7b, bottom left); (c and d) Cystoid macular oedema well shown on FFA (7c) but not seen on spectral OCT (7d)

Macular ischemia is best detected using FFA and has to be looked for in case of severe fundus inflammation such as Behçet's uveitis^9^,^10^ [Figure 7a and b].

#### Cystoid macular edema (CMO)

CMO is visible by funduscopy if it is sufficiently pronounced. Until recently, FFA was, however, recommended to have a precise image of the extent of CMO. Two grading systems of CMO have been put forward by the groups of Miyake and Yanuzzi.^11^,^12^ More recently, optical coherence tomography (OCT) has been used routinely for the evaluation of CMO. In the field of inflammatory diseases, however, FFA still has its place and should at least be performed in parallel with OCT at presentation of the patient, the follow-up being performed by repeated OCTs. Nevertheless, some of the inflammatory CMOs may not have any translation on OCT and are well identified by FFA [Figure 7c and d].

### Sub retinal space

#### Retinal pooling of retinal origin

**Figure 8:**
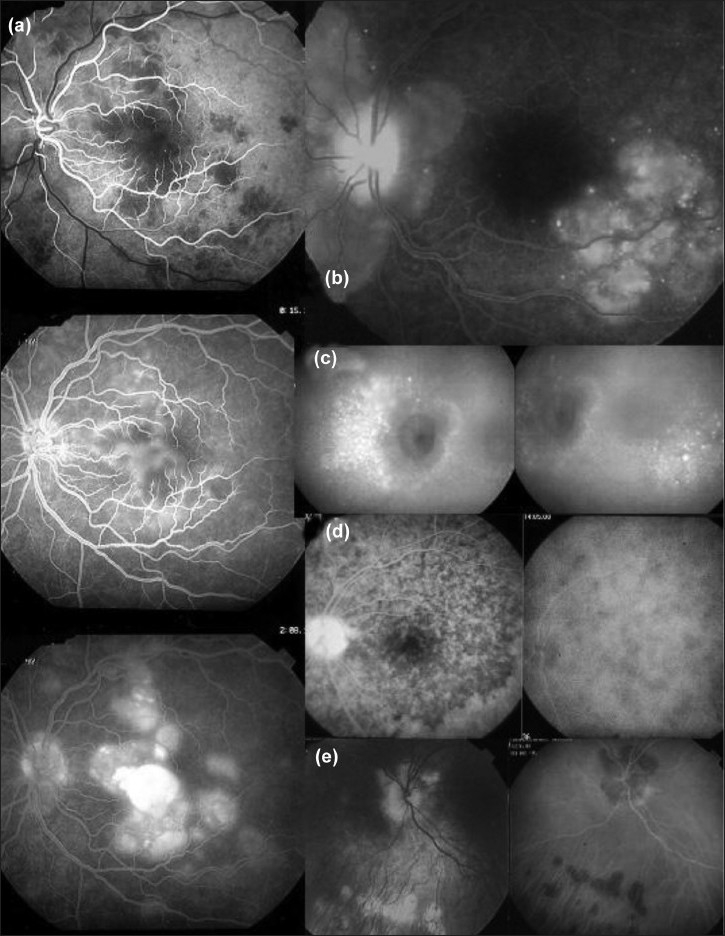
FFA signs: Intraretinal pooling, exudative retinal detachment and atrophy; (a) FFA shows choriocapillaris non perfusion (top) in a patient diagnosed as APMPPE; gradually there is intraretinal and sub retinal pooling (5a, two bottom pictures) due to extrusion of fluorescein coming from the inner retinal capillaries in response to outer retinal ischemia due to choricapillaris non perfusion; (b) FFA exudative retinal detachment with hyperfluorescent pinpoints where choroidal leakage is occurring in a case of VKH disease; (c) Same fundus frame on ICGA showing hyperfluorescence of ERD as well as hyperfluorescent pinpoints-; (d) FFA shows papillitis and mottled RPE with well defined limits between diseased and healthy RPE (high water marks) in a case of VKH. Left picture is an ICG frame of the same area showing numerous persisting HDDs indicating active disease; (e) Chorioretinal atrophy, late hyperfluorescent on FFA and hypo fluorescent on ICGA

Inflammation of the choriocapillaris mostly manifests by non perfusion with the consequence that the outer retina becomes ischemic which in turn produces leakage from reacting inner retinal capillaries. In some of the inflammatory choriocapillaropathies (formerly classified under the term of white dot syndromes), the process can be very pronounced as in some cases of APMPPE (acute posterior multifocal placoid pigment epitheliopathy) causing massive intraretinal and sub retinal pooling of fluorescein. Any other disease causing choroidal ischemia can cause similar pooling [Figure 8a].

#### Exudative retinal detachment of choroidal origin

Sub retinal fluid can also originate directly from the choroid such as in VKH (Vogt-Koyanagi-Harada) disease, where massive primary choroidal inflammation spills over to the retina and causes profuse leakage of liquid through the retinal pigment epithelium (RPE) forming exudative retinal detachments (ERD). Other diseases that can produce ERD include sympathetic ophthalmia and posterior scleritis [Figure 8b and c].

### Retinal pigment epithelium

#### Inhomogeneity of background fluorescence (mottled RPE)

Exudative retinal detachments produce RPE changes characterized by loss of RPE cells causing small areas of window defects as well as clumping of cells causing masking effect. This alternation of loss and clumping of RPE gives a mottled aspect on early angiographic frames due to increased transmission of background choroidal fluorescence (window effect) in some areas together with decreased transmission of choroidal fluorescence (blo*ckin*g effect). Very often, the limits of the ERDs are well identified on FFA by a demarcation line between the affected and normal RPE called high water marks [Figure 8d].

#### Hyperfluorescent pinpoints

The leaking points where fluid passes from the choroid into the subretinal space accumulate a large amount of fluorescein dye and appear brightly hyperfluorescent on FFA (as well as on ICGA), which is explained by the deposition of fluorescein at this transit points [Figure 8b and c].

### Chorioretinal atrophy

Chorioretinal atrophy can be limited to the absence of the choriocapillaris-RPE complex or be a complete atrophy including the choroidal stroma. In the first case, the area is characterized by early hypofluorescence with large stromal choroidal vessels that are hyperfluorescent and well visible on a dark background.

In case of complete atrophy, the whole area is dark on early frames becoming hyperfluorescent on late frames reflecting the bare sclera that is impregnated with fluorescein, which easily diffuses in tissues due to its small size. Atrophic areas on the reverse appear hypofluorescent on ICGA as the large ICG molecular complex is not diffusing and does not impregnate the bare sclera [Figure 8e].

### Choriocapillaris (up to 60″)

Unless the RPE screen is very dark, as in patients of African ancestry, the choriocapillaris fluorescence of the fluorescein dye can be seen despite the presence of the RPE in the early frames of fluorescein angiography when fluorescence is massive. These early frames can show areas of hypofluorescence due to choriocapillaris non perfusion. Alert clinicians understood and adequately interpreted these signs as due to choriocapillaris inflammation causing non perfusion well before ICGA became available and Deutman clearly indicated that disease in APMPPE was primarily due to the choriocapillaris and not the RPE.^12^ Indeed, because the time of access to the choroid is limited with FFA, it is not possible to tell whether this hypofluorescence is due to perfusion delay or complete non perfusion of the choriocapillaris. This can only be shown by ICGA when choriocapillaris is accessible during the whole angiographic sequence; and the interpretation of FFA signs by Deutman for diseases such as APMPPE (and other white dot syndromes) were indeed confirmed by ICGA findings^12^,^13^ [Figure 8a].

### Choroidal inflammatory neovascularization

**Figure 9:**
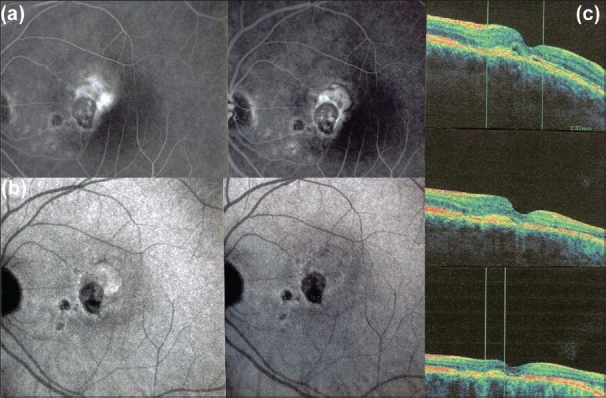
FFA (ICGA) signs: Inflammatory chorioretinal neovascularization. 8a. FFA shows leaking and staining in area adjacent to toxoplasmic scar, before intravitreal Avastin(R) (left picture) and after (right picture); 8b. Same lesion before (L) and after (R) seen by ICGA. 6c. Same lesion seen by OCT, before (top picture) and after Avastin (two bottom OCTs)

Inflammatory choroidal neovessels are mostly type 2 membranes and, therefore, readily accessible to FFA, being above the RPE. Occult choroidal neovessels are also detected by FFA showing progressive diffuse leakage in the macular area. In both these situations, complementary ICGA is, however, recommended. On one hand it can delineate occult membranes and furthermore, in case of CNV associated with a chorioretinal scars, ICGA allows to differentiate between a recurrent inflammatory focus and CNV, the former being early hypofluorescent whereas the latter appears hyperfluorescent since early angiographic frames. ICGA is also mandatory in case of CNV associated with a choriocapillaritis such as multifocal choroiditis, where it shows the extent of occult choriocapillaris non perfusion (iceberg constellation or complex) and hence the danger for CNV development^14^ [Figure 9].

## THE PRINCIPLES OF INDOCYANINE GREEN ANGIOGRAPHY

### The Molecule: Indocyanine green

**Figure 10:**
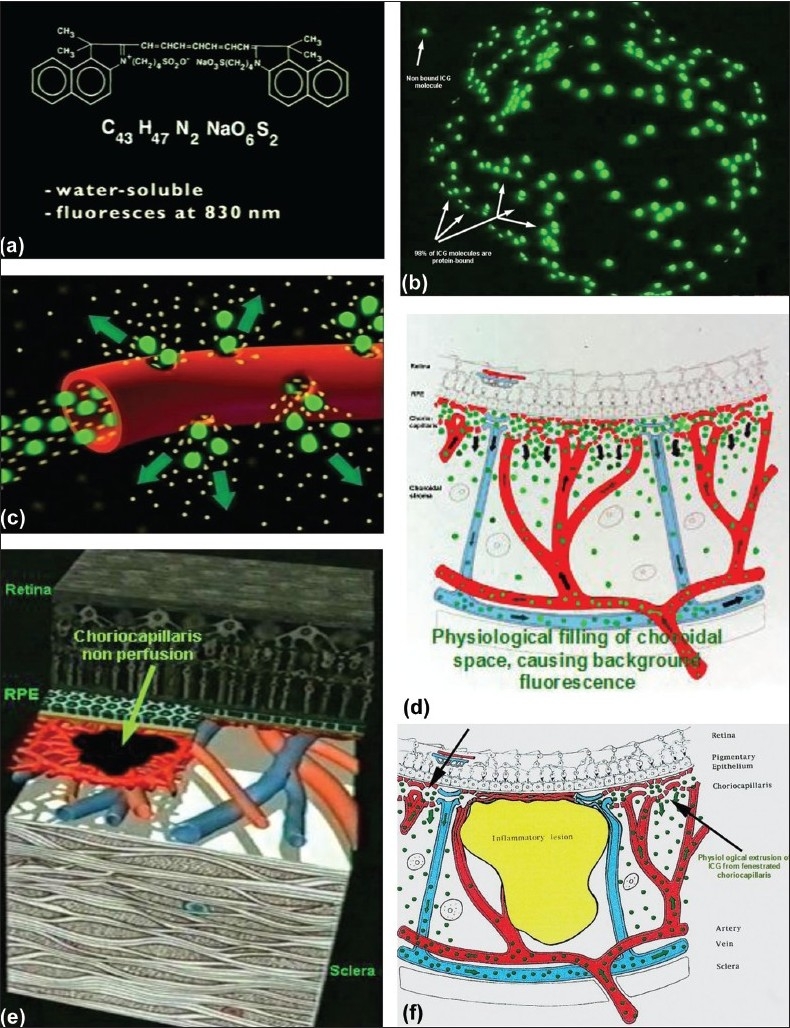
ICG angiographic principles The ICG molecule (775 daltons) (9a) is 98% protein bound forming a large macromolecular ICG-protein complex (> 58'000 daltons) (9b) that extrudes freely through the large fenestrations of the choriocapillaris (9c) progressively impregnating the choroidal stroma (9d).This physiological fluorescence is impaired by two mechanisms producing hypofluorescent ICGA lesions: (1) choriocapillaris non perfusion (9e) occurring in inflammatory choriocapillaropathies and (2) mass effect due to space occupying lesions such as inflammatory foci here shown as the full-thickness stromal granuloma on (9f) explaining the hypofluorescence seen on ICGA

Indocyanine green fluoresces at 830 nm and therefore gives access to the choroidal vascular structures through the retinal pigment epithelium. The molecular weight difference between ICG (775 daltons) and fluorescein (354 daltons) molecules does not account for the specific angiogram pattern obtained with ICG as compared with fluorescein. Besides the different wavelength at which ICG fluoresces, the crucial difference between these two fluorescing molecules comes from their binding affinity to proteins.^15^,^16^ The ICG molecule is nearly completely protein bound and predominantly so to large sized proteins (lipoproteins).^15^ Fluorescein leaks readily from slightly inflamed retinal vessels with minor damage to the blood-retinal barrier and readily impregnates tissues, whereas only major damage to retinal vessels allows ICG to leak.^17^ In the choroid, however, ICG leaks unimpaired but slowly from the fenestrated choriocapillaris^8^ [Figure 10c and d]. During recirculation, more and more ICG is entrapped in the choroidal tissue as the ICG-protein complex is only slowly reabsorbed into the circulation. Gradual impregnation of the choroid occurs with time causing intermediate and late choroidal background fluorescence. This choroidal impregnation by ICG fluorescence is disturbed by choroidal inflammatory lesions, causing areas of decreased or absent fluorescence surrounded by increased fluorescence due to leakage of large choroidal vessels. It is this alteration of the slow choroidal impregnation process that is the main parameter studied in ICGA performed for posterior uveitis.

### Standard ICG angiographic protocol for inflam matory diseases

A standard ICGA protocol to analyze choroiditis has been designed.^18^ The angioraphic procedure comprises three main phases; the early phase up to two to three minutes showing superimposed retinal and choroidal large vessels and incipient exudation of the dye through the choriocapillaris into the choroidal stroma; the intermediate phase at about 10 minutes shows maximum choroidal stromal background fluorescence and; the late phase at about 28–32 minutes shows wash-out of the dye from the general circulation with the large choroidal vessels appearing dark against the background stromal fluorescence.^18^

### Principles for the interpretation of ICGA in uveitis^18^

When analyzing ICG in posterior inflammatory disorders, crucial differences with fluorescein angiography interpretation have to be borne in mind to correctly analyze the images obtained.^19^ During initial circulation, ICG is comparable to fluorescein showing the passage through arteriovenous compartments except that it shows superimposed retinal and choroidal circulations. The difference occurs during recirculation time when ICG is progressively leaking out from the fenestrated choriocapillaris, gradually and physiologically impregnating the whole choroidal thickness.

This process can be altered in two ways that can be associated in the same disease; either there is a decreased fluorescence or an increased fluorescence.

#### ICG hypofluorescence

The impregnation of the choroidal space can be decreased or absent [Figure 11a] (1) by a decrease of the physiological extrusion of the ICG from the choriocapillaris (non perfusion or hypo perfusion, confluent geographic aspect) [Figure 12] or (2) by the impairment of the filling of the choroidal tissue by the ICG molecule because of the presence of space-occupying lesions (inflammatory foci, round even in size and regularly distributed) [Figure 10]. The latter lesions are hypofluorescent in the intermediate angiographic phase. If they remain hypofluorescent in the late phase this signifies that the inflammatory lesion occupies the whole thickness of the choroidal stroma. When lesions become isofluorescent in the late angiographic phase, inflammation causes only partial thickness infiltrates. Therefore, in ICGA performed for inflammatory disorders, the main information is obtained less from the analysis of the early circulatory phase than from the analysis of the altered pattern of the filling of the choroidal space.

**Figure 11:**
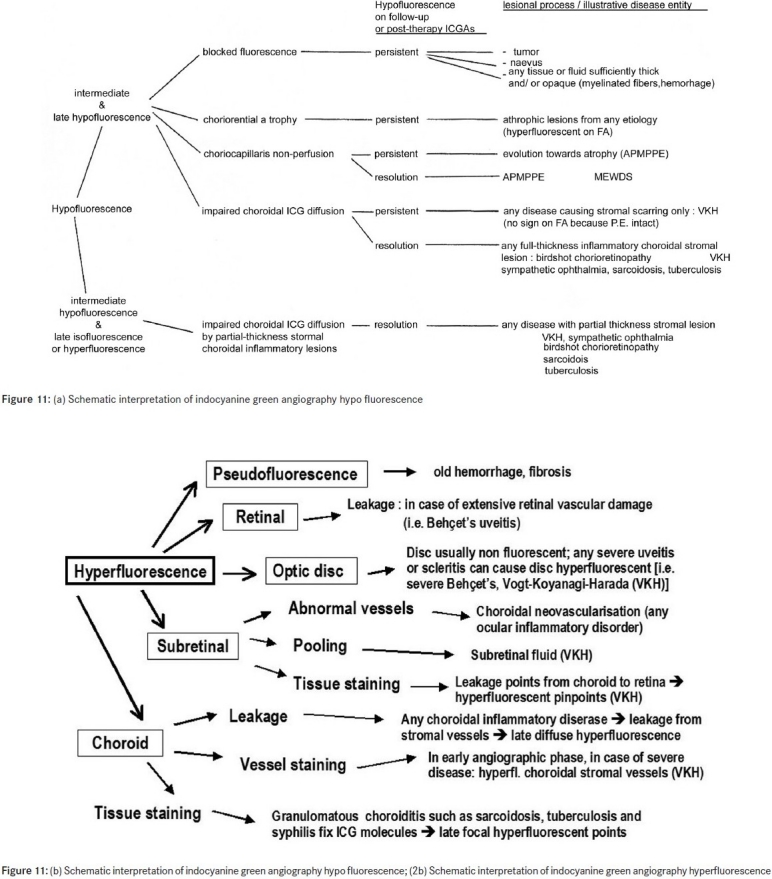
(b) Schematic interpretation of indocyanine green angiography hypofluorescence; (2b) Schematic interpretation of indocyanine green angiography hypofluorescence

**Figure 11a:**
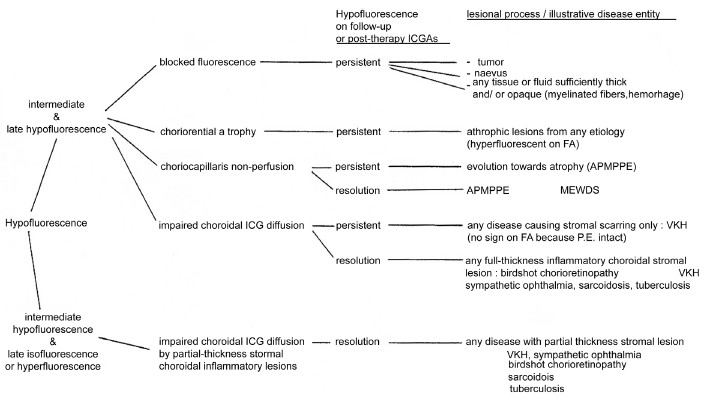
(a) Schematic interpretation of indocyanine green angiography hypofluorescence

**Figure 12:**
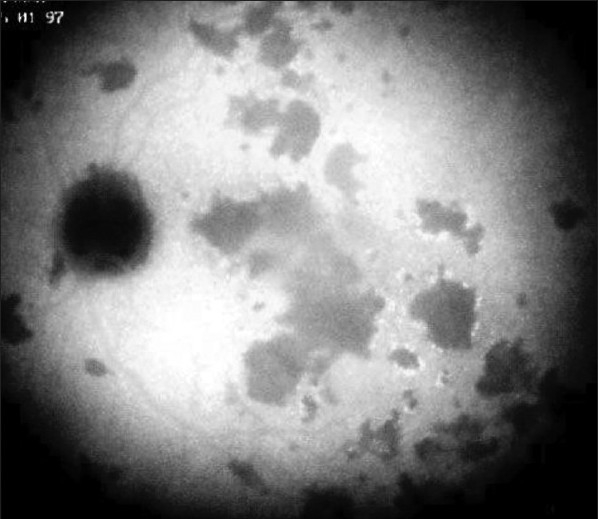
ICGA signs of choriocapillaris non perfusion Areas of patchy or geographic ICGA hypo fluorescent areas of variable sizes present in the early, intermediate and late angiographic phases, in a case of APMPPE, corresponding to confluent plaques of deep fundal discoloration. They often leave atrophic areas as seen in serpiginous choroiditis in the convalescent phase

(Originally published and reproduced from Ophthalmology 1998;105:432–40.)

#### ICG hyperfluorescence

Impregnation of the choroidal space can be enhanced [hyperfluorescence; Figure 11] by increased leakage from the larger choroidal vessels, which adds to the physiological background fluorescence. The vessels appear fuzzy in the intermediate time frames and extrusion of the dye from large vessels causes late diffuse hyperfluorescence [Figure 14].

In case of presence of inflammatory foci in the choroidal stroma, hyperfluorescence is associated with hypofluorescent dark dots due to inflammatory infiltrates.

Hyperfluorescence occurs in two relevant situations, (1) diffuse late phase hyperfluorescence due to leakage from precapillary or larger non fenestrated choroidal vessels and (2) disc hyperfluorescence indicating severe inflammation.

In most cases, unlike in fluorescein angiography where pathology produces hyperfluorescence, the lesions in ICGA are mostly seen in a negative dark pattern due to impaired physiological choroidal fluorescence.

### Fluorescein versus indocyanine green angiography

When analyzing ICGA, it is of utmost importance to make abstraction in most inflammatory situations of two factors that are important in the interpretation of fluorescein angiograms, namely, blockage and window defect [Table 1]. Because infrared fluorescence can be seen through structures that are a screen for visible light, blockage has to be considered only if the interfering structures in front of the choroid are sufficiently thick and/or heavily pigmented. Similarly, the notion of window defect does not usually apply for ICGA as the retinal pigment epithelium generally does not act as a screen as in fluorescein angiography.

**Table 1:** Two main indocyanine green angiography derived lesional mechanisms determine the classification of choroiditis

Choriocapillaris inflammation (primary inflammatory choriocapillaropathies)
MEWDS/AIBSE
APMPPE
Multifocal choroiditis/PIC
Serpiginous choroiditis
Rare entities: AMN, AZOOR
Stromal inflammation (stromal choroiditis) further subdivided into 2 categories
Primary obligatory stromal choroiditiis
Vogt-Koyanagi-Harada disease
Sympathetic Ophthalm Birdshot choriortinopathy
Stromal choroiditis as a random location of a systemic disease
Sarcoidosis
Tuberculosis
Syphilis
Other infectious choroiditides

### Clinico-pathologic-angiographic correlations

The pathologic process at the origin of the ICGA images we see have been verified histopathologically for some of the diseases such as the primary stromal choroiditides Vogt-Koyanagi-Harada disease, sympathetic ophthalmia and birdshot chorioretinopathy, as well as the choroidal lesions of sarcoidosis, while others can still only be hypothesized, needing ICGA-clinico-pathologic correlations.

### Relevance of indocyanine green angiography in ocular inflammatory diseases

Indocyanine green angiography showed occult choroidal lesions not shown by fundoscopy and/or fluorescein angiography in 100% of patients with a well-established diagnosis known to involve the choroid and these findings had an essential impact either on diagnosis or management in 12.3% of these cases, stressing the importance of ICGA for the proper management of most inflammatory process of the back of the eye.^20^

## PRACTICAL USE OF ICGA IN UVEITIS

Indocyanine green angiography is principally useful and has allowed imaging access to the choroidal compartment, which has been poorly utilized to date. In addition to showing often missed choroidal involvement in every day practice, it has allowed to understand choroidal inflammation, classifying choroiditis according to the structure that is preponderantly or initially involved and not simply according to fundus appearance of lesions. At the present stage of our knowledge, there seem to be at least two main mechanisms of inflammation of the choroid.^17^

### Primary inflammatory choriocapillaropathies (White dot syndromes)

This first group of diseases, formerly, mostly included in the inadequate term of “white dot syndromes” results from inflammation at the level of the choriocapillaris causing areas of choriocapillaris non perfusion and its ischemic consequences, both at the level of the choroid and the outer retina, depends on the choriocapillaris for oxygen and nutrients. Acute posterior multifocal placoid pigmentary epitheliopathy (APMPPE) is a disease typically illustrating this type of choroidal inflammation.

#### Angiographic signs in inflammatory choriocapillaro pathies^21^,^22^

Indocyanine green angiographic signs in inflammatory choriocapillaropathies are well determined and have contributed to the recognition of the common mechanism in a group of entities formerly classified as “white dot syndromes”

The following ICGA signs have to be looked for:

1. The hallmark sign of inflammatory choriocapillaropathy is patchy or geographic ICGA hypofluorescent areas of variable sizes present in the early, intermediate and late angiographic phases but usually more clearly visible on the late frames, indicating choriocapillaris non-perfusion or hypo perfusion [Figure 15].
2. Complete or partial regression of the ICGA hypo fluorescence or absence of regression in the convalescent phase: The areas remaining hypofluorescent in the convalescent phase represent chorioretinal atrophy and correspond to areas of window effect and masking effect on fluorescein angiography.
3. In diseases with a progressing course such as serpiginous choroiditis, ICGA can show diffuse choroidal hyperfluorescence at the edges of the progressing lesions in areas having no translation on fundoscopy or fluorescein angiography [Figure 14].

While the ICGA signs are quite uniform, the FA angiographic signs depend on the severity and extension of the choriocapillaris non perfusion and on the outer retinal damage.

1. On FA, there is early hypofluorescence showing the choriocapillaris non perfusion identified on ICGA.
2. Depending on the severity of the choriocapillaris, non perfusion seen on ICGA, late FA frames either show no hyperfluorescence (for instance in mild MEWDS), discrete patchy late hyperfluorescence seen in MEWDS or extensive late hyperfluorescence seen in APMPPE [Figure 15].
  To understand the genesis of the FA signs it is important to be aware that late FA fluorescence is coming from retinal vessels overlying areas of ischemic outer retina that present increased permeability in response to the ischemia due to choriocapillaris non perfusion.
3. In the convalescent phase, there is a delayed regression of FA signs (hyperfluorescence and staining) as compared to the regression of ICGA signs. In case of chorioretinal atrophy window effect and masking effect due to chorioretinal scarring are seen.

Any severe inflammation in an adjacent structure to the choriocapillaris (retina or choroidal stroma) can cause inflammation at the level of the choriocapillaris and produce similar angiographic signs. In that situation we speak of secondary inflammatory choriocapillaropathy.

### Stromal choroiditis

In the second group of diseases, the primary mechanism is the development of inflammatory foci, mostly granulomatous at the level of the stroma appearing hypofluorescent on ICGA, usually associated with inflammation of larger non-fenestrated stromal vessels appearing on ICGA as fuzzy vessels in the intermediate phase followed by diffuse late choroidal hyperfluorescence. Vogt-Koyanagi-Harada disease or birdshot chorioretinopathy are typical illustrations of this type of choroidal inflammation. The mechanism is completely different from the first type of disease, but these conditions have been included by some authors in the “white dot syndromes”.^23^

#### Angiographic signs in stromal choroiditis

Since the availability of ICGA it has become possible to investigate stromal choroidal inflammatory disease in a more subtle way and detect lesions even at an early, often subclinical stage of evolution not accessible so far to investigational tests.^24^

The primary lesion of stromal choroiditis is the inflammatory, mostly granulomatous focus. Depending on the extension of the granuloma, full-thickness or partial thickness, the ICGA image will be influenced. In contrast to primary inflammatory choriocapillaropathies, the mechanism of stromal choroiditis has been proven with the help of anatomo-clinical-angiographic correlations. Histopathology has been available since some time for Vogt-Koyanagi-Harada disease, sympathetic ophthalmia and sarcoidosis and recently granulomas have been found in an autopsy case of birdshot chorioretinopathy.^25^–^27^

**Figure 13:**
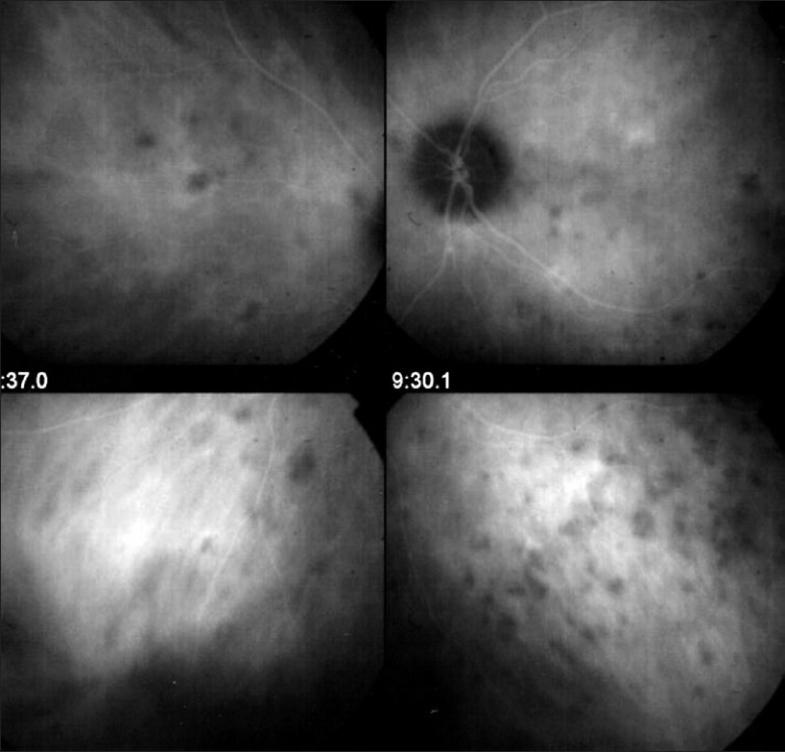
Stromal inflammatory foci/granulomas in Vogt-Koyanagi-Harada disease Typical even regularly distributed hypofluorescent evenly sized dark dots indicating numerous choroidal granulomas

**Figure 14:**
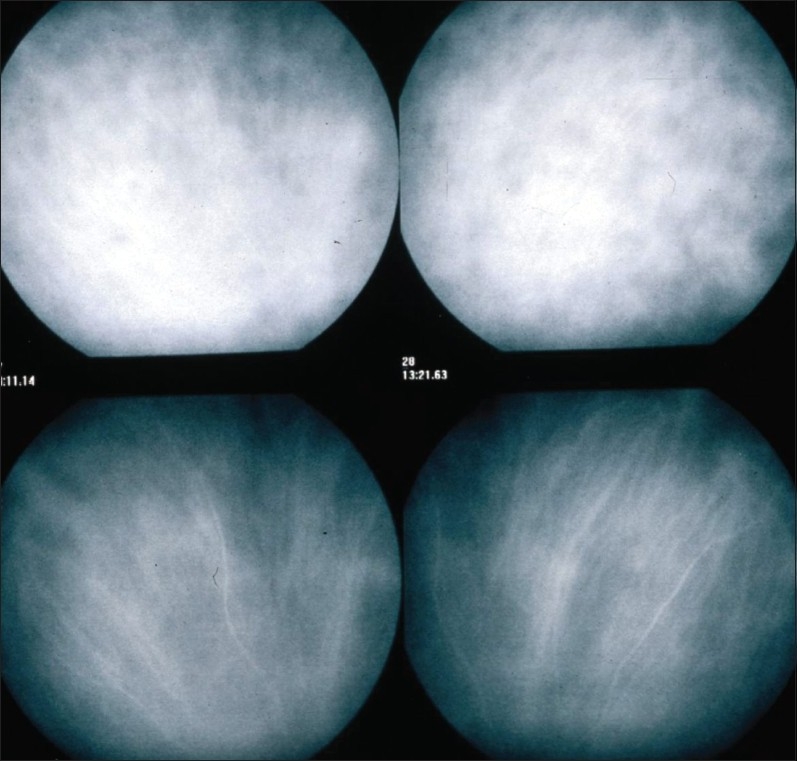
Choroidal stromal vasculitis in Vogt-Koyanagi-Harada disease. Hypo fluorescent dark dots surrounded by indistinct fuzzy choroidal vessels (top frames) showing normalization after only 3 days of pulse intravenous corticosteroid injections (bottom frames)

Different kinds of pathologic behavior have to be distinguished to better understand the lesions seen by fundus examination and FA and ICG angiography. At least two lesional events can take place in the choroidal stroma. The first group of diseases includes those entities with the choroidal stroma as the elective target of the inflammatory process. They can be called the primary obligatory choroiditis and include Vogt-Koyanagi-Harada disease, sympathetic ophthalmia and birdshot chorioretinopathy. Their lesions are characterized by multiple even sized regularly distributed lesions on ICGA accompanied by leaking hyperfluorescent fuzzy choroidal stromal vessels [Figures 13 and 14]. The second group of diseases includes systemic inflammatory or infectious diseases that can involve the choroid by chance, including sarcoidosis, tuberculosis and syphilis. Unlike the first group the size of the lesions are uneven and distributed more at random [Figure 17].

**Figure 15:**
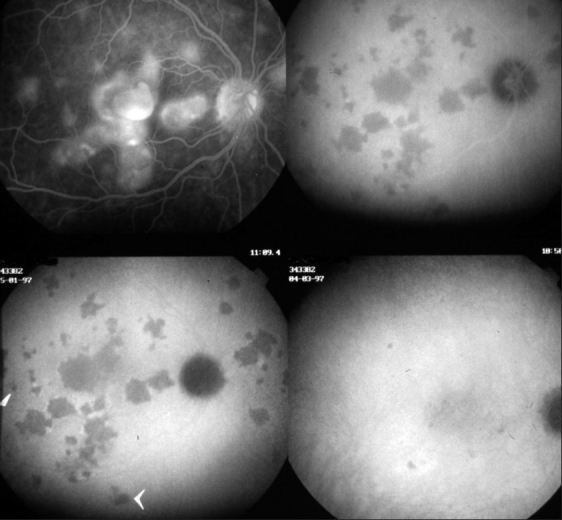
Choriocapillaris non perfusion in a case of APMPPE/AMIC Choriocapillaris non perfusion is shown by patchy geographic areas of hypofluorescence in the intermediate phase of angiography (top right picture) and in the late angiographic phase (bottom left picture) that resolve almost completely in the convalescent stage of disease 2 months later (bottom right picture). The late fluorescein frame on the top left shows hyperfluorescence, corresponding to the ICG areas of choriocapillaris non perfusion that can only be explained by leakage from the capillaries of the inner retina in response to ischemic signals from the outer retina

**Figure 16:**
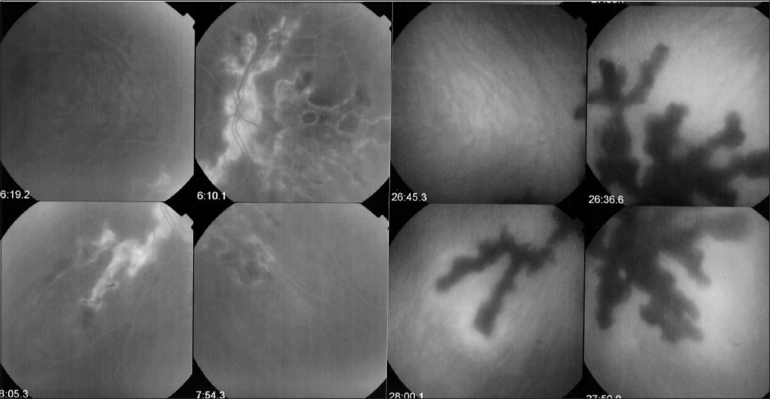
Diffuse perilesional choroidal hyperfluorescence in subclinically progressing serpiginous choroiditis. ICGA (right quartet of pictures) shows many more involved areas than shown by fluorescein angiography (left quartet of pictures) and shows hyperfluorescence around progressing lesions

**Figure 17:**
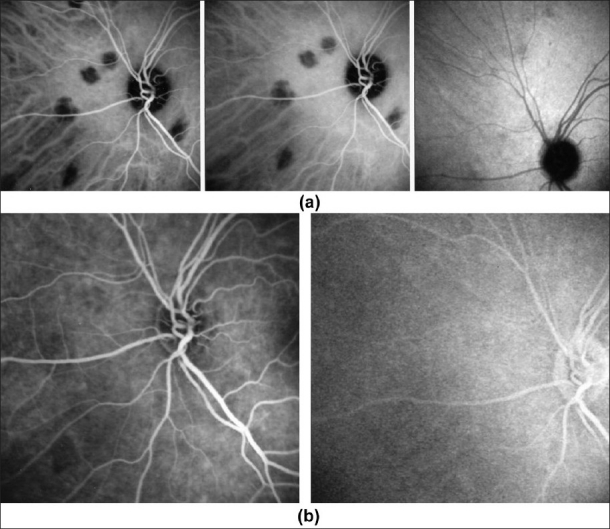
Choroiditis in Sarcoidosis (15a) Unevenly sized randomly distributed lesions; (15b) well seen in the intermediate ICG angiographic phase (15a, middle and left frames) and disappearing in the late phase (right frame), indicating that these presumed granuloma are of partial thickness not filling the stroma from sclera to choriocapillaris. The lesions are hardly visible on fluorescein angiography

The specific ICGA signs that can be seen in diverse proportions according to the type of disease on which this classification relies are the following:

1. Even, regularly distributed ICGA hypofluorescent dots in case of birdshot chorioretinopathy, Vogt-Koyanagi-Harada disease and sympathetic ophthalmia or diversely sized hypofluorescent areas in case of sarcoidosis or tuberculosis present in the early and intermediate phase remaining hypofluorescent in the late phase (full thickness inflammatory foci) or becoming isofluorescent in the late phase in case of partial thickness inflammatory foci, indicating a mass effect.^18^
2. Around the inflammatory lesions, seen as hypofluorescent dark dots, the larger choroidal vessels lose their normal aspect and appear fuzzy indicating choroidal vasculitis that allows pathologic extrusion of the ICG-complex at the origin of late diffuse choroidal hyperfluorescence^18^ [Figure 12].

### Additional indocyanine green angiography signs

Apart from the signs that lead to exploring choroidal inflammation, whether at the level of the choriocapillaris or at the level of the choroidal stroma, additional ICGA signs have been reported.

ICGA was shown to demonstrate pure choroidal vasculitis without choroidal foci (hypofluorescent dark dots) in acute initial attacks of inflammation in Behçet's disease. Subsequently, when patients are under systemic therapy choroidal involvement can no longer be detected.^28^,^29^

The optic disc is usually non fluorescent in ICGA angiography. In case of severe inflammatory disease, disc hyperfluorescence is a characteristic finding usually present in all types of hyper acute diseases such as initial attacks of Behçet's uveitis and VKH disease, representing also a parameter to monitor the effect of therapeutical intervention. In a group of VKH patients, it was shown to regress within one month after introduction of systemic therapy.^30^ ICGA was also shown to be useful in distinguishing reactivation of a retinal focus of toxoplasmosis at the border of a scar versus development of CNV which, instead of being hypofluorescent in the early angiographic phase, presents as early hyperfluorescent, thus allowing elimination of the possibility of reactivation of the inflammatory focus.

### Indocyanine green angiography implications in the practical care of the uveitis patient

#### Precise assessment of choroidal inflammatory involvement

The choroid is the starting point of the inflammation in many diseases including MEWDS, APMPPE, multifocal choroiditis, serpiginous choroiditis, VKH, sympathetic ophthalmia and birdshot chorioretinopathy and participates in the inflammatory process in many other diseases such as sarcoidosis, tuberculosis, syphilis, toxoplasmosis, posterior scleritis and many more which have extensively been analyzed by ICGA.^31^–^41^ A reliable inventory of fundus inflammatory involvement can, therefore, only be performed with the help of ICGA. It contributes essentially to the assessment of disease extension in those conditions involving the choroid.

**Figure 18:**
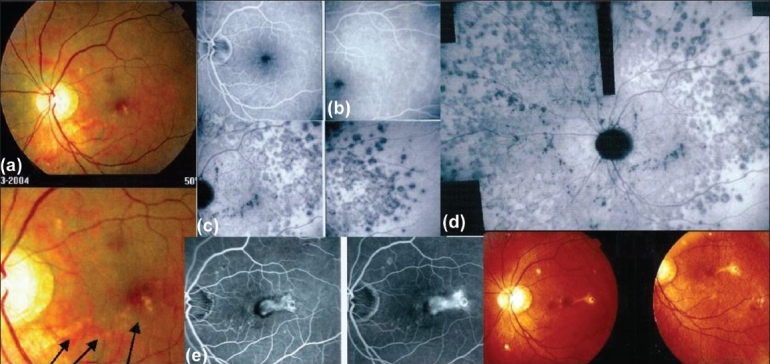
Multifocal choroiditis (the iceberg effect or constellation). Fundus pictures show minimal signs limited to faint depigmentation (16a, black arrows) and FFA shows absolutely no abnormalities (16b), whereas ICGA shows extensive choriocapillaris involvement with large geographic areas of non perfusion (16c and 16 d) which we now know to be a risk factor for CNV, which the patient developed one year later as seen on FFA (16e) and fundus images (bottom right)

**Figure 19:**
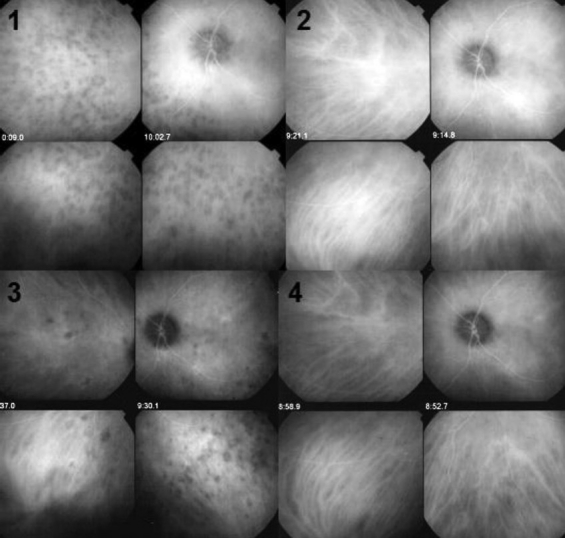
ICGA guided treatment of VKH disease. 17.1. VKH at presentation: Numerous hypofluorescent dark dots indicating granulomatous foci in the choroid as well as fuzzy choroidal vessels indicating choroidal vasculitis. Following intravenous pulse steroid therapy followed by high dose oral steroids HDDs disappear completely and choroidal vessels regain a normal pattern (17.2) Upon tapering there is recrudescenc of subclinical choroidal disease (17.3.), that prompts reincrease of oral steroid therapy and introduction of azathioprine. After slow tapering of steroid therapy first followed by tapering of azathioprine over 2 years the patient remains recurrence free.(17.4)

In some diseases such as MEWDS, only choroidal signs can be present and in others the clinically apparent part of the disease such as in multifocal choroiditis is only the peak of the iceberg (iceberg constellation) and the preponderant part of the disease can only be followed by ICGA [Figure 18]. In diseases originating from the choroid such as VKH disease, the only way to detect subclinical disease is to perform ICGA [Figure 19].

#### Diagnostic contribution of indocyanine green angiography

**Figure 20:**
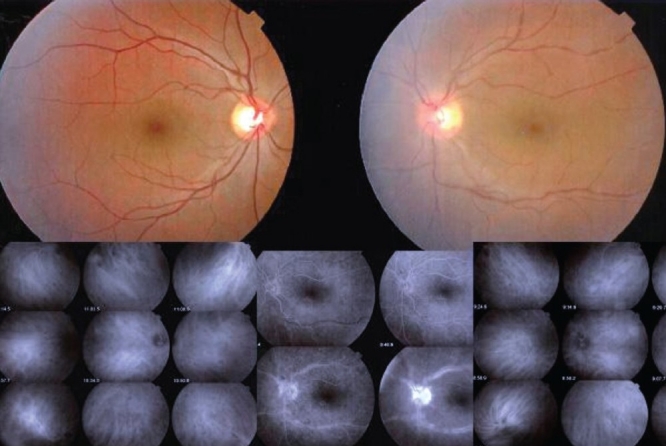
The essential role of ICGA for early diagnosis of birdshot retinochoroiditis. Patient in his mid-forties presenting with blurred vision OS due to the presence of vitritis (top right). FFA shows retinal vasculitis OS of large veins and small vessels (bottom middle). The right eye seems not affected (top left). However, ICGA shows numerous hypofluorescent dark dots on the left (bottom right) but also on the right (bottom left), typical for birdshot retinochoroidopathy

Unlike for FFA, it is not rare that ICGA gives the essential contribution that leads to the diagnosis. This can be the case for patients with MEWDS that have minimal translation in fundus signs or FFA signs. Early birdshot chorioretinopathy can only be diagnosed using ICGA as the early lesions are hypofluorescent dark dots indicating subclinical granulomas that can only be detected by ICGA. These ICGA lesions often precede the typical oval shaped depigmented “birdshot” lesions by several months or even years [Figure 20].

#### Indocyanine green angiography monitoring of disease evolution and response to therapy

As choroidal lesions can only be detected by ICGA, it is obviously the recommended modality to monitor the evolution and to evaluate the impact of treatment on choroidal inflammatory process.^30^ In case of VKH disease, clinical disease, meaning inflammation involving extra choroidal structures accessible to fundus observation, to OCT and FFA, can be followed by classical means. However, once clinical disease is under control, it has been shown that subclinical disease is ongoing, resulting in almost 100% of cases in sunset-glow-fundus (SGF) the witness of ongoing disease destroying choroidal pigment. It was recently shown that ICGA guided treatment of VKH could avoid evolution towards SGF when treating subclinical disease shown by ICGA^42^ [Figure 19].

## SUMMARIZING REMARKS

Fluorescein angiography is performed routinely in inflammatory ocular diseases, which is probably justified to make a good evaluation of inflammation and use it as a follow-up parameter for superficial fundus structures. Except for the evaluation of retinal vessels, it generally does not add essential information to clinical examination and OCT. In contrast, ICGA often furnishes additional information, otherwise undetectable, on the choroidal compartment. Several choroidal diseases such as inflammatory choriocapillaropathies (MEWDS, multifocal choroiditis and APMPPE and others) present with the “iceberg constellation” meaning minimal signs seen on funduscopy or FFA with extensive choroidal involvement. Therefore, except for conditions such as pars planitis or Behçet's disease where choroiditis is absent or insignificant, dual FFA and ICGA should be performed for the assessment and follow-up of posterior uveitis, if angiographic analysis is deemed necessary. A dual FFA/ ICGA angiographic scoring method has recently been proposed for that very purpose.^43^

## CONCLUSION

Gradually, the precision of assessment of intraocular inflammation and its complications have become sharper in new technical developments such as ICGA, which go beyond the information given by more established methods; integrated with the choice of complementary investigations. In contrast to other new investigational modalities such as OCT, which represent an improved visualization of lesions often already accessible with other means such as funduscopy or FA, ICGA shows additional lesions, mainly in the choroid, usually not accessible or demonstrated by other means. The choroid is responsible for posterior inflammation in an equal if not higher proportion of cases when compared to the superficial structures as the retina. Therefore, in cases of suspected choroidal involvement, ICGA is as or more important than FA; dual ICGA/FA angiography is highly recommended in such cases.
